# A Machine Learning–Based Prognostication Model Enhances Prediction of Early Hepatic Encephalopathy in Patients With Noncancer-Related Cirrhosis: Multicenter Longitudinal Cohort Study in Taiwan

**DOI:** 10.2196/71229

**Published:** 2025-08-06

**Authors:** Hsin-Yu Chen, Yiu-Hua Cheng, Wei-Chung Yeh, Yi-Chuan Chen, Yi-Wen Tsai

**Affiliations:** 1 Department of Family Medicine, Keelung Chang Gung Memorial Hospital, Keelung, Taiwan; 2 School of Medicine, College of Medicine, Chang Gung University, Taoyuan, Taiwan; 3 Department of Family Medicine, New Taipei Municipal TuCheng Hospital, New Taipei, Taiwan; 4 Department of Family Medicine, Linkou Chang Gung Memorial Hospital, 5 Fuxing Street, Guishan District, Taoyuan, Taiwan, 886 33281200 ext 2482; 5 Graduate Institute of Medical Sciences, College of Medicine, National Defense Medical University, Taipei, Taiwan

**Keywords:** hepatic encephalopathy, machine learning model, cirrhosis, noncancer-related liver diseases, prognostication

## Abstract

**Background:**

Hepatic encephalopathy (HE) contributes significantly to mortality among patients with liver cirrhosis. Early prediction of HE is essential for clinical decision-making, yet remains challenging—particularly in noncancer-related cirrhosis due to the unpredictable disease course.

**Objective:**

This study aimed to develop a novel machine learning (ML) model to improve early prediction of HE in patients with noncancer-related cirrhosis.

**Methods:**

A multicenter, retrospective cohort study was conducted from January 2010 to December 2017 across all Chang Gung Memorial Hospital branches in northern, middle, and southern Taiwan. We applied several ML models to evaluate HE predictability and compared their performance in the training dataset and testing dataset. Optimal sensitivity and specificity were determined using the Youden index. The best ML model was interpreted by the Shapley Additive Explanations plot.

**Results:**

A total of 5878 patients with cirrhosis were included in the analysis, of whom 1187 (20.2%) subsequently developed HE. Compared to the non-HE group, patients with HE were older (median age 55, IQR 46‐65 vs median age 54, IQR 44‐66 years; *P*=.04) and had higher rates of hepatitis B virus infection (351/1187, 30% vs 961/4691, 20.5%; *P*<.001), alcohol use (540/1187, 45.5% vs 1512/4691, 32.2%; *P*<.001), sepsis (393/1187, 33.1% vs 792/4691, 16.9%; *P*<.001), and mortality (425/1187, 35.8% vs 502/4691, 10.7%; *P*<.001), along with distinct laboratory abnormalities reflecting liver dysfunction. Among the ML algorithms evaluated, the extreme gradient boosting algorithm demonstrated the highest predictive accuracy, achieving an area under the curve (AUC) of 0.86 (95% CI 0.83‐0.88) in the testing dataset. This performance was significantly superior to that of the neural network (AUC 0.79, 95% CI 0.76‐0.81; *P*<.001), support vector machine (AUC 0.77, 95% CI 0.73‐0.80; *P*<.001), and the model for end-stage liver disease score (AUC 0.74, 95% CI 0.71‐0.77; *P*<.001). Using a probability threshold of 0.25, the extreme gradient boosting model demonstrated a sensitivity of 72% (95% CI 0.67‐0.77), specificity of 80% (95% CI 0.78‐0.82), a positive predictive value of 48% (95% CI 43-53), and a negative predictive value of 92% (95% CI 90-94) in the testing set. Comparable performance was observed in the training dataset, with a sensitivity of 80% (95% CI 0.77‐0.83), specificity of 81% (95% CI 0.80‐0.82), and a negative predictive value of 94% at the same threshold. The most influential predictive variables identified by the model included serum ammonia, aspartate transaminase, alanine transaminase, prothrombin time, and serum potassium.

**Conclusions:**

We developed a novel ML model for predicting HE in patients with noncancer-related cirrhosis. This model provides a practical guide to help physicians and these patients in shared decision-making regarding treatment strategy, with the ultimate goal of improving clinical care and reducing the burden of HE-related morbid complications.

## Introduction

Complications of chronic liver cirrhosis affect millions of individuals worldwide, which causes a tremendous burden and health expenditure for these patients and their families [1,2]. Among these cirrhosis-related complications, hepatic encephalopathy (HE) has emerged as a severe neuropsychiatric manifestation contributing to substantial morbidity and mortality, increased hospitalization, medical costs, readmission rates, and impaired health-related quality of life [1-5]. HE represents a major clinical concern for physicians to necessitate timely intervention and to monitor and mitigate the adverse outcomes in patients with cirrhosis. However, the current sensitivity and specificity of diagnostic tools used in HE vary depending on the different testing methods used across studies and settings [6,7]. Additionally, due to the diverse stages and unpredictable trajectories in patients with cirrhosis, formidable challenges in disease management and prognostication remain critical for clinicians to resolve, especially for patients with noncancer-related cirrhosis [6].

Etiologies of noncancer-related cirrhosis vary, including hepatitis B virus (HBV) and hepatitis C virus (HCV) infection, alcohol-related liver disease, and nonalcoholic steatohepatitis. A global statistics estimation revealed a higher prevalence of noncancer-related cirrhosis than that of cancer-related cirrhosis [1]. Moreover, a recent longitudinal analysis from National Health Research Institutes data in Taiwan between 2000 and 2017 reported only about 14.2% palliative resource use in the year prior to death in patients with noncancer-related terminal disease compared with 60.9% use in patients with terminal cancer in 2017 [8], which highlights that the use of palliative care services within the year preceding death in patients with a noncancer-related terminal illness was far less than that in patients with cancer-related terminal illness. The National Health Insurance system in Taiwan is a nationwide program launched by the Taiwanese government that covers over 96% of Taiwan’s population registered in the census for over 6 months. The administrative datasets are maintained electronically by the National Health Research Institutes and the National Health Insurance Administration of Taiwan [9-11]. Patients with end-stage liver disease (ESLD) without hepatocellular carcinoma are significantly less likely to receive inpatient specialist palliative care and to have a lower use rate compared with those with hepatocellular carcinoma [12,13]. The distress and extreme pain experienced by patients with noncancer-related ESLD is comparable to that of individuals with cancer-related terminal illnesses such as lung and colon cancer [14]. However, given the complexities of the etiology and unpredictability of disease trajectories in patients with noncancer cirrhosis, these gaps in unmet clinical needs between patients with cancer and noncancer-related cirrhosis underscore the importance of the early recognition and prognosis of cirrhosis complications, such as HE. Taken together, these data emphasize the fact that the need for prognostication and early prediction for incident HE in patients with noncancer-related cirrhosis is important.

Despite advances in medical therapeutics and supportive care, the accurate prediction and early detection of HE remain a formidable clinical task. Traditional prognostic tools, such as the model for end-stage liver disease (MELD) score, offer valuable insights into disease severity and prognosis but lack the predictive accuracy needed for optimal clinical decision-making [6]. Moreover, MELD scores do not account for factors such as inflammation, ammonia levels, or other metabolic disturbances. Furthermore, tests for HE that rely on psychometric performance can be directly influenced by social determinants of health, smoking, diabetes, and alcohol use, which are factors that can vary by region [7].

Machine learning (ML) algorithms have emerged as promising tools for predictive modeling and risk stratification in complex medical conditions. By leveraging vast datasets and advanced computational techniques, ML algorithms have the potential to identify subtle patterns and predictive features that may elude traditional statistical methodologies.

In this study, we aimed to develop a novel ML approach model to enhance the prognostication and early prediction of incident HE in patients with noncancer-related cirrhosis with the goal to help physicians in shared decision-making for treatment strategy as well as to improve patient care and reduce their distress from morbid complications.

## Methods

### Study Design and Participants

We conducted a multicenter retrospective cohort study involving 8615 patients diagnosed with liver cirrhosis from 3 academic medical centers and 5 community hospitals (Taipei, Linkou, Keelung, Taoyuan, Chiayi, Kaohsiung, Lovers Lake, and Yunlin) of Chang Gung Memorial Medical Foundation in northern, middle, and southern Taiwan between 2010 and 2017. Datasets were collected from the electronic medical record (EMR) system built by the Chang Gung Medical Foundation. Patients with chronic liver disease with more than 2 visits in the EMR system who were diagnosed with cirrhosis defined using the International Classification of Diseases (ICD)-9 and ICD-10 codes (Table S1 in Multimedia Appendix 1) by clinical physicians with or without ESLD-related complications, such as HE, hepatorenal syndrome, ascites, spontaneous bacterial peritonitis, and esophageal varices, were enrolled. HE was defined as having ICD-9 code 572.2 and ICD-10 codes K76.82, B15.0, B16.0, B16.2, B19.0, G93.4, and G94.3 recorded in the EMR system. The exclusion criteria were patients who were younger than 18 years of age, those who had been diagnosed with malignant neoplasms of the liver (ICD-9 code: 155.0 or ICD-10 code: C22-C22.09) or HE at the time of enrollment, or those who had only a one-time-visit record before being lost to follow-up.

### Ethical Considerations

This study was conducted in accordance with the ethical standards of the Declaration of Helsinki, and the protocol was approved by the ethics institutional review board of Chang Gung Memorial Hospital, Taiwan (approval: 201801291B0C504). The institutional review board approved the waiver of informed consent because of the deidentification of all health data collected from the EMR system. The data were collected from patients treated at Chang Gung Memorial Hospital between 2010 and 2017.

### Data Collection and Definitions

Datasets were collected from the EMR system. Individuals meeting the inclusion criteria and whose laboratory data were available within enrollment were included in this study. The laboratory measurements collected for ML included prothrombin time (PT), activated partial thromboplastin time, the international normalized ratio (INR), C-reactive protein (CRP), albumin, ammonia, alanine transaminase (ALT), aspartate transaminase (AST), serum bilirubin, γ-glutamyl transferase, serum bilirubin, creatinine, blood urea nitrogen (BUN), sodium (Na), potassium (K), lactate, uric acid, total cholesterol, triglyceride, fasting plasma glucose, high-density lipoprotein cholesterol levels, low-density lipoprotein cholesterol levels, white blood count, red blood cell, hemoglobin, and platelet. The MELD score was calculated according to the following formula [15]: 3.78×log_e_(bilirubin [mg/dL])+11.2×log_e_(INR)+9.57×log_e_(creatinine [mg/dL])+6.43.

### Data Preprocessing and ML Modeling

This study followed the TRIPOD (Transparent Reporting of a Multivariable Prediction Model for Individual Prognosis or Diagnosis) reporting guidelines to ensure methodological transparency and reproducibility. A completed TRIPOD checklist is provided as Checklist 1. The datasets were randomly divided into training and testing datasets at a 3:1 ratio. We implemented random forest (RF), neural network, support vector machine (SVM), and extreme gradient boosting (XGBoost) for modeling in the training dataset. The models were built and evaluated using R (version 4.0.1; R Foundation for Statistical Computing) with the *xgboost* package (version 1.4.2). The testing dataset was performed for internal validation. Missing data were addressed through median imputation, where the median value was used to replace any unavailable or null value. Model training was completed in 2024 based on structured clinical data from EMR records.

The prediction target was the probability of developing HE, with the model output ranging from 0 to 1. This continuous output was transformed into a binary classification.

Model training used 10-fold cross-validation with hyperparameter tuning, but no alignment strategies (such as reinforcement learning or preference optimization) were used. Inference was based on structured variable inputs, and no text generation, prompt engineering, or large language models were involved in the modeling process.

### Feature Selection and Imbalanced Data

To identify significant candidate features while eliminating noisy or redundant ones that could lead to an inefficient, impractical, or overfitting model, we used a wrapper method using the Boruta algorithm to rank the predictive features of HE. For practical purposes, we repeated the Boruta algorithm 300 times and selected the top 20 ranked features. To avoid the bias toward the majority class and enhance the overall accuracy and performance of the model, we used the synthetic minority over-sampling technique for imbalanced datasets.

### Performance Evaluation and Interpretation

We assessed the area under the receiver operating characteristic curve (AUC-ROC) to discriminate the performance of the ML models. We further adopted the Youden index to evaluate the best corresponding cutoff sensitivities and specificities for the optimal performance of the ML algorithm. Then, we analyzed the model using the Shapley Additive Explanations (SHAP) plot to disclose the impact of the features on the output of the model prediction.

### Statistical Analysis

Continuous variables were expressed as the median (IQR), and categorical variables by frequencies and percentages. ML modeling was performed using the statistical software package R (version 4.0.1) with the *Boruta* [16] and *Caret* packages [17]. Prediction models were compared using R with the DeLong method of the *pROC* package [18]. All statistic assessments were evaluated at a 2-sided α level of .05.

## Results

### Overview

**Figure 1.**
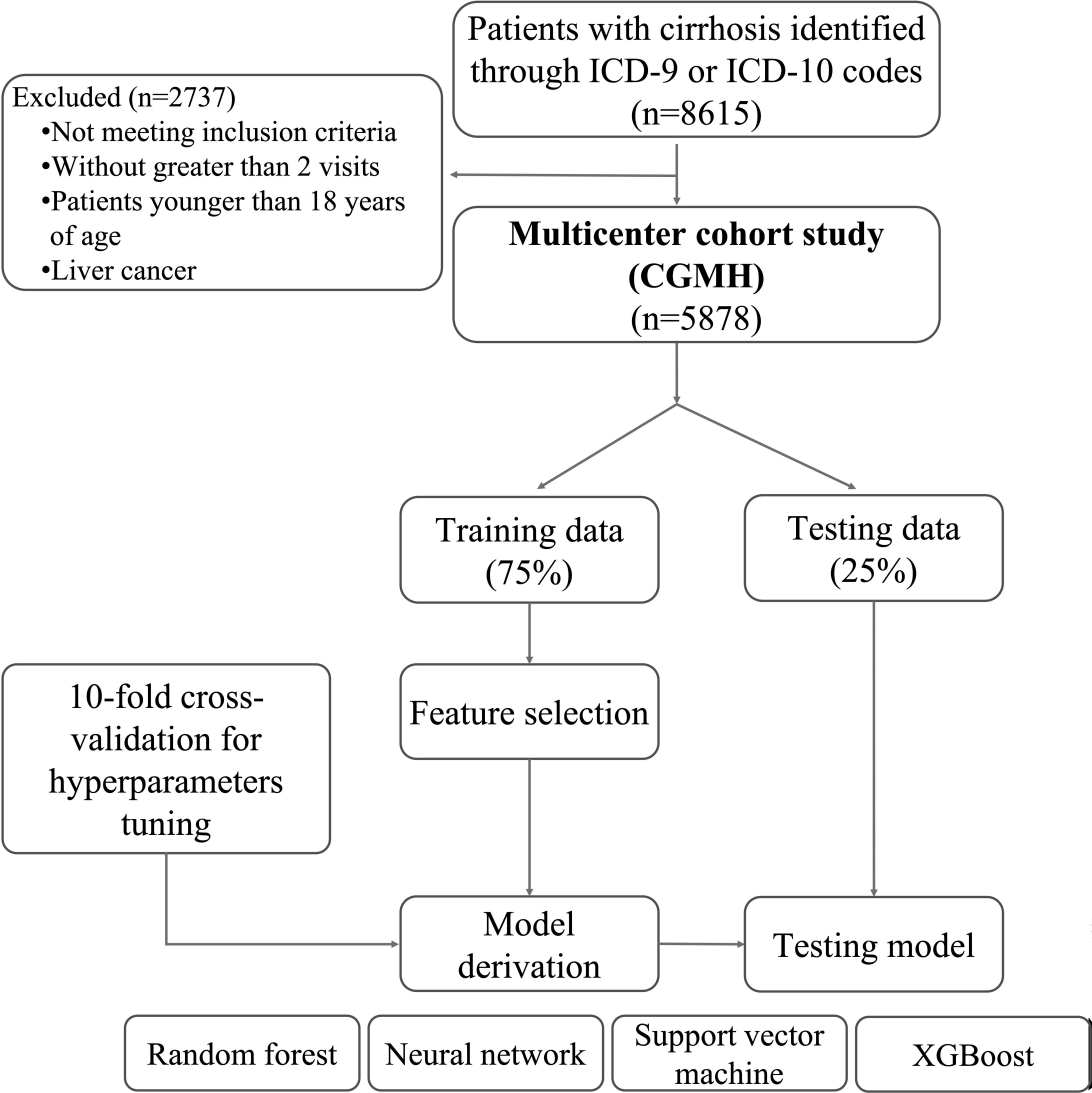
Flowchart of the enrollment process. ICD: International Classification of Diseases; CGMH: Chang Gung Memorial Hospital; XGBoost: extreme gradient boosting.

A total of 8615 consecutive patients who had been diagnosed as cirrhosis, with or without ESLD-related complications, were enrolled in this study. These patients were identified from the EMR system in 3 academic medical centers and 5 community hospitals of Chang Gung Memorial Medical Foundation in Taiwan, between January 1, 2010, and December 31, 2017. After applying the exclusion criteria—including patients with fewer than 2 visits, those younger than 18 years of age, and those with liver cancer—5878 patients with cirrhosis were eligible for analysis, including 1187 with HE and 4691 without HE. Figure 1 shows a flowchart of the enrollment process.

### Baseline Characteristics of the HE and Non-HE Groups in Patients With Noncancer-Related Liver Cirrhosis

The median age was slightly older in the HE group compared to the non-HE group (median 55, IQR 46-65 vs median 54, IQR 44-66 years; *P*=.04). There were no significant differences in sex or HCV infection between the groups. Specifically, 845 of 1187 (71.2%) patients in the HE group and 3227 of 4691 (68.8%) in the non-HE group were male, 342 of 1187 (28.8%) patients in the HE group and 1464 of 4691 (31.2%) in the non-HE group were female (*P*=.11). HCV infection was present in 211 of 1187 (17.8%) and 882 of 4691 (18.8%) patients in the HE and non-HE groups, respectively (*P*=.40). In contrast, the prevalence of HBV infection was higher in the HE group (351/1187, 30%) than in the non-HE group (961/4691, 20.5%; *P*<.001). Alcohol use was reported in 540 of 1187 (45.5%) patients with HE versus 1512 of 4691 (32.2%) patients without HE (*P*<.001). Sepsis occurred in 393 of 1187 (33.1%) patients with HE compared to 793 of 4691 (16.9%) without HE (*P*<.001). Mortality was also significantly higher in the HE group, with 425 (35.8%) deaths versus 502 (10.7%) deaths in the non-HE group (*P*<.001). In laboratory comparisons, patients in the HE group had significantly lower platelet counts, prolonged PT, activated partial thromboplastin time, and INR, higher serum levels of white blood count, ALT, AST, total bilirubin, direct bilirubin, γ-glutamyl transferase, ammonia, lactate, BUN, and creatinine, and lower levels of serum albumin, potassium, sodium, total cholesterol, high-density lipoprotein, and hemoglobin. No significant differences were observed in serum uric acid, low-density lipoprotein cholesterol, or blood sugar levels between the 2 groups.

Overall, in our multicenter retrospective longitudinal analysis, the HE group showed a greater tendency to have coagulopathy, hepatorenal syndrome, and hypoalbuminuria, which may have been the result of malnutrition or sarcopenia in the patients with noncancer-related liver cirrhosis. The baseline demographic and laboratory characteristics between the HE and non-HE groups are shown in Table 1.

**Table 1.** Comparisons of baseline characteristics between non-HE^a^ and HE groups (n=5878).

Characteristics	HE (n=1187)	Non-HE (n=4691)	*P* value^b^^,^^c^
Age (years), median (IQR)	55 (46-65)	54 (44-66)	.04
Sex, n (%)			.11
Female	342 (28.8)	1464 (31.2)	
Male	845 (71.2)	3227 (68.8)	
Sepsis, n (%)	393 (33.1)	792 (16.9)	<.001
Hepatitis B virus, n (%)	351 (30)	961 (20.5)	<.001
Hepatitis C virus, n (%)	211 (17.8)	882 (18.8)	.40
Alcohol use, n (%)	540 (45.5)	1512 (32.2)	<.001
Death, n (%)	425 (35.8)	502 (10.7)	<.001
International normalized ratio, median (IQR)	1.56 (1.30-2.00)	1.20 (1.10-1.46)	<.001
Prothrombin time, median (IQR)	16.5 (13.7-21.2)	13.1 (11.4-15.7)	<.001
Activated partial thromboplastin time, median (IQR)	40 (33-52)	31 (29-37)	<.001
C-reactive protein, median (IQR)	22 (10-46)	17 (4-53)	<.001
Albumin, median (IQR)	2.60 (2.30-2.90)	3.00 (2.50-3.64)	<.001
Alanine transaminase, median (IQR)	37 (23-66)	31 (19-54)	<.001
Aspartate transaminase, median (IQR)	73 (45-131)	48 (29-90)	<.001
Total bilirubin, median (IQR)	4 (2-12)	1 (1-3)	<.001
Direct bilirubin, median (IQR)	3.4 (0.9-10.9)	0.3 (0.2-1.0)	<.001
γ-Glutamyl transferase, median (IQR)	66 (32-153)	60 (21-163)	.02
Ammonia, median (IQR)	129 (91-182)	99 (72-138)	<.001
Blood urea nitrogen, median (IQR)	20 (11-42)	15 (10-24)	<.001
Creatinine, median (IQR)	0.97 (0.63-1.83)	0.82 (0.62-1.18)	<.001
Lactate, median (IQR)	23 (16-45)	18 (12-48)	.004
Sodium, median (IQR)	138.0 (134.0-141.0)	139.0 (136.0-141.0)	<.001
Potassium, median (IQR)	3.60 (3.10-4.10)	3.80 (3.40-4.20)	<.001
Uric acid, median (IQR)	5.50 (3.48-8.20)	6.00 (4.60-7.50)	.08
Total cholesterol, median (IQR)	125 (98-155)	146 (117-175)	<.001
High-density lipoprotein cholesterol, median (IQR)	25 (14-33)	31 (20-42)	<.001
Low-density lipoprotein cholesterol, median (IQR)	67 (50-92)	76 (55-102)	.07
Sugar, median (IQR)	131 (99-184)	135 (101-192)	.60
White blood count (1000), median (IQR)	6.8 (4.6-10.1)	6.1 (4.2-8.7)	<.001
Red blood cell, median (IQR)	3.12 (2.71-3.60)	3.53 (2.98-4.19)	<.001
Hemoglobin, median (IQR)	9.70 (8.50-11.00)	10.40 (8.90-12.40)	<.001
Hematocrit, median (IQR)	29 (26-32)	31 (27-37)	<.001
Mean corpuscular volume, median (IQR)	93 (86-99)	90 (85-96)	<.001
Mean corpuscular hemoglobin, median (IQR)	31.5 (29.2-33.8)	30.4 (28.3-32.4)	<.001
Mean corpuscular hemoglobin concentration, median (IQR)	33.85 (32.90-34.80)	33.50 (32.60-34.40)	<.001
Red blood cell distribution width, median (IQR)	17.7 (15.9-20.3)	16.0 (14.5-18.4)	<.001
Platelet (1000), median (IQR)	78 (52-115)	106 (63-179)	<.001

a HE: hepatic encephalopathy.

b *P *values calculated using Pearson chi-square test for categorical variables [19].

c *P *values calculated using the Wilcoxon rank sum test for nonnormally distributed continuous variables [20].

### XGBoost Exhibited the Best Discriminatory Performance Compared With All ML Algorithms and the MELD Score in Both the Training and Validation Datasets

**Figure 2.**
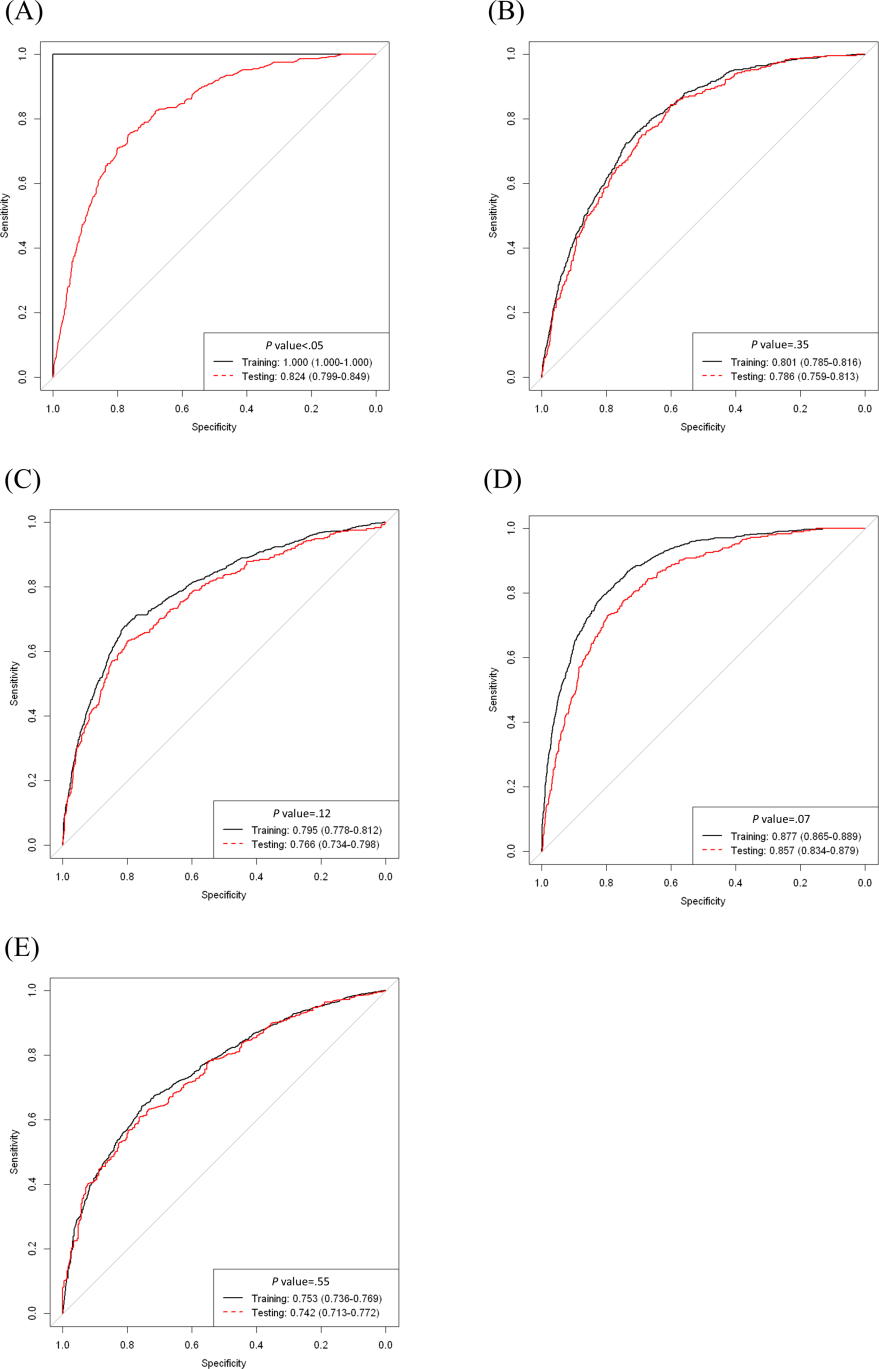
Performance of the machine learning models: (A) random forest, (B) neural network, (C) support vector machine, (D) extreme gradient boosting, and (E) model for end-stage liver disease score.

We further performed the 4 AutoML algorithms, including RF, neural network, SVM, and XGBoost, to compare their performance in terms of predicting HE in patients with noncancer-related ESLD using the training dataset in the EMR system compared to the MELD score and performed further validation using the testing dataset. We first demonstrated that XGBoost exhibited the best discriminatory performance (AUC-ROC 0.86, 95% CI 0.83‐0.88) compared with RF (AUC-ROC 0.82, 95% CI 0.80‐0.85), neural network (AUC-ROC 0.79, 95% CI 0.76‐0.81), SVM (AUC-ROC 0.77, 95% CI 0.73‐0.80), and the MELD score (AUC-ROC 0.74, 95% CI 0.71‐0.77; Tables2  3 and Figure 2). The cutoff value by Youden index for XGBoost for discriminating HE was 0.25 (training dataset: sensitivity 80%, 95% CI 0.77‐0.83; specificity 81%, 95% CI 0.80‐0.82; positive predictive value [PPV] 0.52, 95% CI 0.49‐0.54; negative predictive value [NPV] 0.94, 95% CI 0.93‐0.95; positive likelihood ratio 4.22, 95% CI 3.91‐4.55; negative likelihood ratio 0.25, 95% CI 0.21‐0.28; and testing dataset: sensitivity 72%, 95% CI 0.67‐0.77; specificity 80%, 95% CI 0.78‐0.82; PPV 0.48, 95% CI 0.43‐0.53; NPV 0.92, 95% CI 0.90‐0.94; positive likelihood ratio 3.64, 95% CI 3.18‐4.16; negative likelihood ratio 0.35, 95% CI 0.29‐0.42; Table 4).

**Table 2.** Performance of the build prediction model in training and testing datasets.

Methods and dataset		Accuracy (95% CI)	Sensitivity (95% CI)	Specificity (95% CI)	AUC-ROC^a^ (95% CI)
Random forest					
	Training	1.000 (0.99‐1.00)	1.00	1.00	1.00 (1.00‐1.00)
	Testing	0.82 (0.80‐0.84)	0.32	0.95	0.82 (0.80‐0.85)
Neural network					
	Training	0.81 (0.80‐0.82)	0.22	0.96	0.80 (0.79‐0.82)
	Testing	0.80 (0.78‐0.82)	0.25	0.94	0.79 (0.76‐0.81)
Support vector machine					
	Training	0.82 (0.81‐0.83)	0.18	0.98	0.80 (0.78‐0.81)
	Testing	0.81 (0.79‐0.83)	0.20	0.97	0.77 (0.73‐0.80)
XGBoost^b^					
	Training	0.85 (0.84‐0.86)	0.42	0.96	0.88 (0.87‐0.89)
	Testing	0.84 (0.82‐0.85)	0.38	0.95	0.86 (0.83‐0.88)
MELD^c^ score					
	Training	N/A^d^	N/A	N/A	0.75 (0.74‐0.77)
	Training	N/A	N/A	N/A	0.74 (0.71‐0.77)

a AUC-ROC: area under the receiver operating characteristic curve.

b XGBoost: extreme gradient boosting.

c MELD: model for end-stage liver disease.

d N/A: not applicable.

**Table 3.** Performance of the build prediction model in training and testing datasets.

Methods	Dataset	Accuracy (95% CI)	Sensitivity (95% CI)	Specificity (95% CI)	AUC-ROC^a^ (95% CI)	*P* value
Random forest	Testing	0.82 (0.80‐0.84)	0.32	0.95	0.82 (0.80‐0.85)	.05
Neural network	Testing	0.80 (0.78‐0.82)	0.25	0.94	0.79 (0.76‐0.81)	<.001
Support vector machine	Testing	0.81 (0.79‐0.83)	0.20	0.97	0.77 (0.73‐0.80)	<.001
XGBoost^b^	Testing	0.84 (0.82‐0.85)	0.38	0.95	0.86 (0.83‐0.88)	—^c^
MELD^d^ score	Training	N/A^e^	N/A	N/A	0.74 (0.71‐0.77)	<.001

a AUC-ROC: area under the receiver operating characteristic curve.

b XGBoost: extreme gradient boosting.

c Not available.

d MELD: model for end-stage liver disease.

e N/A: not applicable.

**Table 4.** Performance of the extreme gradient boosting model for hepatic encephalopathy prediction in training and validation datasets (Youden index=0.25).

	Training set	Testing set
Sensitivity (95% CI)	0.80 (0.77-0.83)	0.72 (0.67-0.77)
Specificity (95% CI)	0.81 (0.80-0.82)	0.80 (0.78-0.82)
Positive predictive value (95% CI)	0.52 (0.49-0.54)	0.48 (0.43-0.53)
Negative predictive value (95% CI)	0.94 (0.93-0.95)	0.92 (0.90-0.94)
Positive likelihood ratio (95% CI)	4.22 (3.91-4.55)	3.64 (3.18-4.16)
Negative likelihood ratio (95% CI)	0.25 (0.21-0.28)	0.35 (0.29-0.42)

Regarding performance in predicting incident HE, XGBoost achieved the best accuracy in the testing dataset compared with RF, neural network, SVM, and the MELD score (XGBoost: accuracy 0.84, 95% CI 0.82‐0.85; RF: accuracy 0.82, 95% CI 0.80‐0.84; neural network: accuracy 0.80, 95% CI 0.78‐0.82; SVM: accuracy 0.81, 95% CI 0.79‐0.83). These findings strongly indicate that our ML-based XGBoost algorithm is a superior tool for predicting HE in patients with noncancer-related cirrhosis in comparison with the other ML models and traditional MELD scores, especially for ruling out disease when the finding returns a negative result; this could be an applicable tool to guide clinicians in palliative care clinical settings (Tables 2-4 and Table S2 in Multimedia Appendix 1).

### Serum Ammonia Was the Foremost Important Feature, Followed by Other Variables, in the ML-Based XGBoost Algorithm for Predicting HE in Patients With Noncancer-Related ESLD

**Figure 3.**
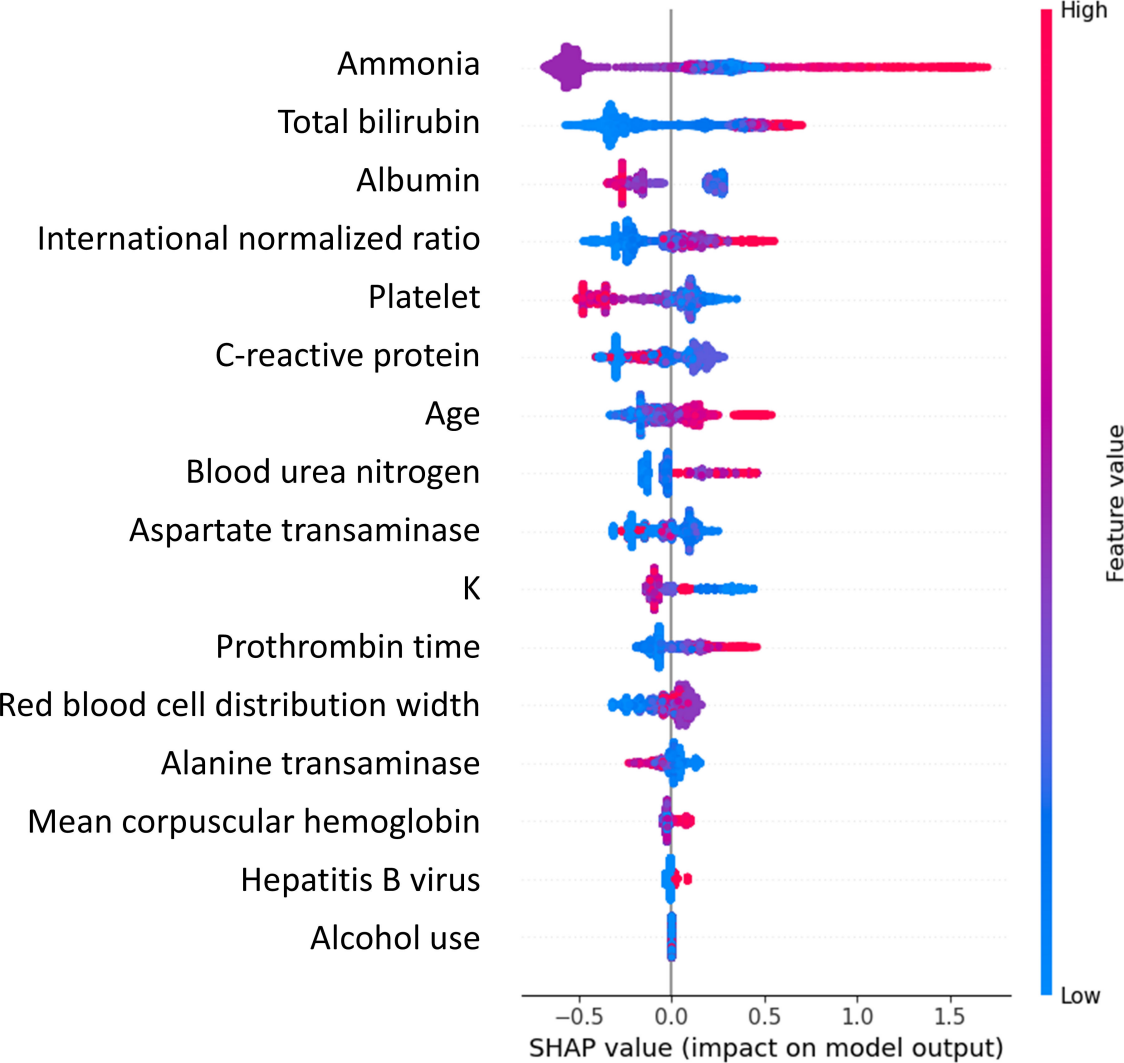
SHAP summary plot to explain the feature importance obtained by the extreme gradient boosting algorithm. Features with higher feature value (red) and positive SHAP value (on the right side) show a positive association, while features with higher feature value (blue) and negative SHAP value (on the left side) show a negative association. K: potassium; SHAP: Shapley Additive Explanations.

Additionally, we constructed a SHAP to reveal the weight of each feature on the outcomes of the trained model’s predictability based on feature importance. We demonstrated the essential features’ contribution to the predictive outcomes, including serum levels of ammonia, total bilirubin, INR, CRP, patient age, total platelet count, BUN, PT, K, AST, albumin, red blood cell distribution width, ALT, mean corpuscular hemoglobin, and history of HBV infection (Table 5). Based on the SHAP values of feature importance, we further disclosed that higher levels of serum ammonia, total bilirubin, INR, older age, BUN, and PT, lower serum levels of albumin and serum K, and lower platelet counts result in a higher probability of HE in patients with noncancer-related ESLD. Furthermore, among the 15 key variables in the AutoML model based on the XGBoost algorithm, ammonia was the most important feature impacting the probability of HE risk, followed by total bilirubin, INR, CRP, age, platelet, BUN, PT, and serum K (Figure 3).

**Table 5.** Feature importance from the extreme gradient boosting model based on Shapley Additive Explanations values.

Rank	Clinical feature
1	Ammonia
2	Total bilirubin
3	Albumin
4	International normalized ratio
5	Platelet
6	C-reactive protein
7	Age
8	Blood urea nitrogen
9	Aspartate transaminase
10	Potassium
11	Prothrombin time
12	Red blood cell distribution width
13	Alanine transaminase
14	Mean corpuscular hemoglobin
15	Hepatitis B virus
16	Alcohol use

Taken together, compared with other ML-based models, such as RF, neural network, and SVM, our ML-based XGBoost algorithm demonstrated the best performance for HE predictability in patients with noncancer-related ESLD. Among the variables in the XGBoost model, serum ammonia levels, together with other feature variables, constituted the most important influence regarding HE predictability in patients with noncancer-related cirrhosis.

## Discussion

### Principal Findings

In our multicenter retrospective longitudinal study, we developed an ML-based prognostic model to improve the early prediction of HE in patients with noncancer-related cirrhosis. Among the evaluated algorithms, the XGBoost model consistently demonstrated superior predictive performance in terms of discriminability, accuracy, and robustness—particularly in ruling out HE with high NPV—across both training and validation datasets.

The choice of XGBoost was driven by its technical advantages over other ML algorithms such as RF, neural network, and SVM. Unlike RF, which may experience overfitting in complex high-dimensional data, XGBoost incorporates regularization techniques that reduce variance and improve generalizability. Additionally, XGBoost handles multicollinearity and missing values more effectively than logistic regression and is computationally efficient for structured tabular data. These characteristics make XGBoost a particularly well-suited algorithm for clinical prediction tasks using EMR datasets. Our novel ML-based XGBoost predictive model is an applicable tool to guide clinicians in shared decision-making of treatment strategy to further improve patient care and reduce their distress from morbid complications in clinical settings of noncancer-related populations.

To enhance model interpretability, we conducted a SHAP analysis to identify and visualize the contribution of individual predictors to the model output. This analysis revealed that serum ammonia is the most important feature, followed by bilirubin, INR, age, and BUN, which are positively associated with increased HE risk; whereas albumin, platelet counts, and serum potassium are negatively associated with HE risk. Notably, serum potassium and transaminases emerged as key predictors of HE—findings that warrant further clinical interpretation. From a pathophysiological perspective, hypokalemia is known to exacerbate HE by increasing renal ammonia production and systemic ammonia load [21,22]. This mechanistic link is supported by our data, where lower serum potassium was associated with increased HE risk. Similarly, elevated AST and ALT levels reflect ongoing hepatic inflammation or necroinflammation, which may impair ammonia detoxification, thereby promoting HE [23]. These markers, therefore, not only reflect liver injury but also serve as early warning signs for impending encephalopathy, reinforcing their clinical utility in risk stratification and monitoring. Furthermore, to our knowledge, this is the first study to predict HE in a Taiwanese adult population using an ML algorithm. Our use of SHAP further enhances model transparency and interpretability—both of which are critical for clinical adoption. SHAP values enable clinicians to understand how individual variables influence predictions, thereby building trust and supporting shared decision-making with patients.

Despite the pathogenesis of HE not being fully understood, an increasing number of studies have shown that various forms of inflammation, such as systemic inflammation, neuroinflammation, endotoxemia, and ammonia-inflammation synergism, significantly contribute to the development of HE [24]. Serum CRP, which is known as an inflammation marker, is associated with the development of HE and can serve as a predictor of adverse outcomes and increased risk of HE in patients hospitalized with liver cirrhosis [25,26]. In our data, the HE group exhibited a significantly increased level of serum CRP compared with the non-HE group (*P*<.001) among patients with noncancer-related ESLD. It is of note that our SHAP for feature importance analysis did not show a strong positive influence of serum CRP levels on the increase in HE risk in patients with noncancer cirrhosis. One of the possible reasons for these different study findings could be that the baseline settings of the populations differed between these 2 studies as well as the fact that the pathogenic etiology of HE in populations without cancer differs significantly from that in patients with terminal cancer. In our dataset, the results could have been influenced by outliers of serum CRP because the median serum CRP level in the HE group was significantly higher than that in the non-HE group. This indicates that CRP still plays an important role in HE occurrence.

### Comparison to Prior Work

Our findings revealed notable differences in baseline characteristics between patients with noncancer-related ESLD with and without HE, highlighting the heterogeneity of populations with cirrhosis and the multifactorial nature of HE development. Specifically, patients with HE exhibited higher prevalences of HBV infection, alcohol use, sepsis, and mortality rates compared with their counterparts without HE. In another study [3], among 49,164 patients diagnosed with HE, 24,183 (49.2%) were affected by alcohol-related cirrhosis, 18,352 (37.3%) had HCV infection, and only 2589 (5.3%) had HBV-related cirrhosis, with some cases showing overlap. These differences may be due to the high prevalence of HBV infection in Taiwan. However, other research indicates a decline in the age-standardized incidence rates for liver cirrhosis due to HBV globally from 1990 to 2019, while the incidence rates for nonalcoholic fatty liver disease, alcohol use, and other causes increased during the same period [2]. This suggests a potential shift in the etiology of cirrhosis.

Importantly, our study demonstrates that the XGBoost algorithm has superior discriminatory performance in predicting HE compared with other ML algorithms and MELD scores. The robust sensitivity, specificity, PPV, NPV, and accuracy of the XGBoost model underscore its potential applicability as a valuable clinical decision support tool for early HE detection and risk stratification in populations with noncancer-related cirrhosis. The MELD score, initially designed to predict survival after elective transjugular intrahepatic portosystemic shunt placement, has been validated for predicting survival across diverse patient cohorts with varying liver disease severity and from different geographic and temporal backgrounds. Moreover, the MELD score is widely used to prioritize liver transplantation, primarily relying on serum bilirubin, creatinine, and INR levels. Although it provides a general assessment of liver function, it may not fully capture the multifactorial nature of HE development. By contrast, the capacity of ML algorithms to manage complex interactions and nonlinear relationships is a significant advantage that can improve the accuracy of prediction models. Our XGBoost model incorporated a broader range of variables, including ammonia, total bilirubin, INR, age, BUN, albumin, platelet count, and serum K, allowing for a more detailed and accurate prediction of HE risk. This approach may offer valuable insights into disease pathogenesis and prognosis.

Recent studies have demonstrated the value of ML in liver disease prognostication. Verma et al [27] stratified patients with acute-on-chronic liver failure into survival-based clusters using integrated ML methods. Malik et al [28] reviewed ML applications in predicting esophageal variceal bleeding, while Müller et al [29] identified early cirrhosis decompensation using ML models. Other studies used neural networks to forecast 1-year mortality after variceal bleeding [30] or liver transplantation [31], emphasizing the trend toward long-term outcome modeling. These findings underscore ML’s expanding role in liver disease prediction. Our study contributes to this field by focusing on early HE prediction in noncancer-related cirrhosis using a transparent and interpretable XGBoost framework. Another regional study conducted on 1256 patients with cirrhosis with unbalanced data used several ML methods, specifically SVM, logistic regression, and CatBoost, to predict HE [32]. Although the weighted RF model emerged with an accuracy of 0.8732 (95% CI 0.8711‐0.8752) and an AUC-ROC of 0.82, in that study, the HE predictability was far inferior to our ML-based XGBoost model. Our XGBoost model significantly outperformed weighted RF, achieving an accuracy of 0.84 (95% CI 0.82‐0.85) and an AUC-ROC of 0.86 (95% CI 0.83‐0.88). It showed advantages on XGBoost. XGBoost uses boosting with regularization to reduce overfitting and improve generalization. It also handles missing data and multicollinearity effectively. XGBoost is more efficient and interpretable for structured clinical data better than SVM and neural networks [33,34]. Additionally, we applied SHAP to identify and visualize the contributions of individual features to the model’s predictions. Its consistent and locally accurate attributions improve interpretability, facilitating clinical trust by clarifying how key variables may influence disease outcomes [35].

### Limitations

While our study contributes to advancing the understanding of HE prediction and prognostication of patients with noncancer-related cirrhosis, several limitations warrant consideration. First, despite our application of the Boruta algorithm for feature selection and the use of the synthetic minority over-sampling technique for imbalanced datasets, retrospective analyses still inherently carried biases related to data completeness, documentation accuracy, and confounding variables. Second, the generalizability of our findings to diverse patient populations and health care settings may be limited. As aforementioned, the different etiology of ESLD may affect model application.

### Future Directions

Building upon the aforementioned limitations, future research should prioritize prospective validation in diverse populations and explore the integration of the predictive model into clinical decision support systems. Moreover, external validation across different institutions and geographic settings is essential to evaluate the model’s generalizability and transportability. Embedding the model into cloud-based platforms or EMR systems may further enhance its accessibility and facilitate real-world clinical implementation. Additionally, regarding the other scoring systems, such as the Bilirubin-Albumin-Beta-Blocker-Statin score and MASQ-HE score developed by Tapper et al [36,37], both include adding medications, such as the use of nonselective β-blockers and statins, in addition to quality of life and physical function, to improve the performance in terms of the predictability of HE risk. Therefore, integrating additional clinical variables may enhance model accuracy for the prediction of HE and thus should be considered in the future development of ML-based models.

### Conclusions

Our ML-based XGBoost model demonstrated superior predictive performance for the early detection and prognostication of HE in patients with noncancer-related cirrhosis. Beyond its predictive accuracy, the model shows promise as a clinically valuable tool for use in terminal care settings, outperforming previously established ML algorithms and conventional prognostic scores such as the MELD score. To the best of our knowledge, this is the first ML-based model specifically developed for prognostication and HE prediction in a Taiwanese adult population with noncancer-related cirrhosis—a subgroup with higher prevalence and more unpredictable disease trajectories compared to patients with cancer-related cirrhosis. By incorporating a broader set of clinical features and leveraging SHAP-based interpretability, our model facilitates more precise risk stratification and may support clinicians in making informed decisions aimed at preventing severe complications and improving patients’ quality of life. Future studies are warranted to externally validate this model in larger and more diverse populations and to assess its clinical utility through prospective follow-up of patient outcomes.

## Supplementary material

10.2196/71229 Multimedia Appendix 1 Supplementary tables.

10.2196/71229 Checklist 1 TRIPOD (Transparent Reporting of a Multivariable Prediction Model for Individual Prognosis or Diagnosis) checklist.

## Abbreviations

ALT: alanine transaminase
AST: aspartate transaminase
AUC: area under the curve
AUC-ROC: area under the receiver operating characteristic curve
BUN: blood urea nitrogen
CRP: C-reactive protein
EMR: electronic medical record
ESLD: end-stage liver disease
HBV: hepatitis B virus
HCV: hepatitis C virus
HE: hepatic encephalopathy
ICD: International Classification of Diseases
INR: international normalized ratio
MELD: model for end-stage liver disease
ML: machine learning
NPV: negative predictive value
PPV: positive predictive value
PT: prothrombin time
RF: random forest
SHAP: Shapley Additive Explanations
SVM: support vector machine
TRIPOD: Transparent Reporting of a Multivariable Prediction Model for Individual Prognosis or Diagnosis
XGBoost: extreme gradient boosting
