# Supplementary material for: A Machine Learning–Based Prognostication Model Enhances Prediction of Early Hepatic Encephalopathy in Patients With Noncancer-Related Cirrhosis: Multicenter Longitudinal Cohort Study in Taiwan
Source: JMIR Med Inform. 2025 Aug 6;13:e71229. doi: 10.2196/71229 (PMC12327908; doi:10.2196/71229)
Supplement: Multimedia Appendix 1 [file medinform-v13-e71229-s001.docx]

Table S1. Diagnosis of cirrhosis in this study by using ICD-9 and ICD-10 code.

|  | **ICD-9 code** | | **ICD-10 code** | |
| --- | --- | --- | --- | --- |
| **Cirrhosis** | 571.2 | Alcoholic cirrhosis of liver | K70.30 | Alcoholic cirrhosis of liver without ascites |
|  | 571.5 | Cirrhosis of liver without mention of alcohol | K70.31 | Alcoholic cirrhosis of liver without ascites |
|  | 571.6 | Biliary cirrhosis | K70.40 | Alcoholic hepatic failure without coma |
|  |  |  | K70.41 | Alcoholic hepatic failure with coma |
|  |  |  | K71.7 | Toxic liver disease with fibrosis and cirrhosis of liver |
|  |  |  | K72.00 | Acute and subacute hepatic failure, without coma |
|  |  |  | K72.01 | Acute and subacute hepatic failure, with coma |
|  |  |  | K72.10 | Chronic hepatic failure, without coma |
|  |  |  | K72.01 | Chronic hepatic failure, with coma |
|  |  |  | K72.90 | Hepatic failure, unspecified, without coma |
|  |  |  | K72.91 | Hepatic failure, unspecified, with coma |
|  |  |  | K74.3 | Primary biliary cirrhosis |
|  |  |  | K74.4 | Secondary biliary cirrhosis |
|  |  |  | K74.5 | Biliary cirrhosis, unspecified |
|  |  |  | K74.60 | Unspecified cirrhosis of liver |
|  |  |  | K74.60 | Other cirrhosis of liver |
| **Cirrhosis complication** | 456.0 | Esophageal varices with bleeding | I85.00 | esophageal varices without bleeding |
|  | 456.1 | Esophageal varices without bleeding | I85.01 | esophageal varices with bleeding |
|  | 456.2 | Esophageal varices in diseases classified elsewhere with bleeding | I85.10 | secondary esophageal varices without bleeding |
|  | 456.21 | Esophageal varices in diseases classified elsewhere without bleeding | I85.11 | Secondary esophageal varices with bleeding |
|  |  |  | I86.4 | Gastric varices |
|  | 567.23 | Spontaneous bacterial peritonitis | K65.2 | Spontaneous bacterial peritonitis |
|  |  |  | K65.0 | Generalized (acute) peritonitis |
|  |  |  | K65.8 | Other peritonitis |
|  |  |  | K65.9 | Peritonitis, unspecified |
|  | 572.2 | Hepatic encephalopathy | K76.82 | Hepatic encephalopathy |
|  |  |  | K70.41 | Alcoholic hepatic failure with coma |
|  | 572.4 | Hepatorenal syndrome | K76.7 | Hepatorenal syndrome |
|  | 789.5 | Ascites | K70.11 | Alcoholic hepatitis with ascites |
|  |  |  | K70.31 | Alcoholic cirrhosis of liver with ascites |
|  |  |  | R18 | Ascites |
| **Hepatic encephalopathy** | 572.2 | Hepatic encephalopathy | K76.82 | Hepatic encephalopathy |
|  |  |  | B15.0 | Hepatitis A with hepatic coma |
|  |  |  | B16.0 | Acute hepatitis B with delta-agent (coinfection) with hepatic coma |
|  |  |  | B16.2 | Acute hepatitis B without delta-agent with hepatic coma |
|  |  |  | B19.0 | Unspecified viral hepatitis with hepatic coma |
|  |  |  | G93.4 | Encephalopathy, unspecified |
|  |  |  | G94.3 | Encephalopathy in diseases classified elsewhere |
| **Hepatocellular Carcinoma** | 155.0 | Malignant neoplasm of liver primary | C22 | Malignant neoplasm of liver and intrahepatic bile ducts |
|  |  |  | C22.0 | Liver cell carcinoma |
|  |  |  | C22.1 | Intrahepatic bile duct carcinoma |
|  |  |  | C22.2 | Hepatoblastoma |
|  |  |  | C22.3 | Angiosarcoma of liver |
|  |  |  | C22.4 | Other sarcomas of liver |
|  |  |  | C22.7 | Other specified carcinomas of liver |
|  |  |  | C22.8 | Malignant neoplasm of liver, primary, unspecified as to type |
|  |  |  | C22.9 | Malignant neoplasm of liver, not specified as primary or secondary |

Table S2. Performance of the eXtreme gradient boosting (XGBoost) model with different cut-off. (Youden index = 0.25)

|  | **Training** | | | **Testing** | | |
| --- | --- | --- | --- | --- | --- | --- |
| **Cut-off** | **Accuracy** | **Sensitivity** | **Specificity** | **Accuracy** | **Sensitivity** | **Specificity** |
| 0 | 0.20 | 1.00 | 0.00 | 0.20 | 1.00 | 0.00 |
| 0.05 | 0.50 | 0.99 | 0.37 | 0.45 | 0.96 | 0.32 |
| 0.10 | 0.65 | 0.96 | 0.57 | 0.61 | 0.90 | 0.54 |
| 0.15 | 0.73 | 0.92 | 0.68 | 0.68 | 0.83 | 0.64 |
| 0.20 | 0.77 | 0.86 | 0.75 | 0.74 | 0.77 | 0.73 |
| 0.25 | 0.81 | 0.80 | 0.81 | 0.79 | 0.72 | 0.81 |
| 0.30 | 0.83 | 0.74 | 0.85 | 0.80 | 0.63 | 0.84 |
| 0.35 | 0.84 | 0.66 | 0.89 | 0.81 | 0.56 | 0.88 |
| 0.40 | 0.85 | 0.58 | 0.92 | 0.82 | 0.52 | 0.90 |
| 0.45 | 0.85 | 0.48 | 0.94 | 0.82 | 0.42 | 0.92 |
| 0.50 | 0.85 | 0.41 | 0.96 | 0.82 | 0.34 | 0.94 |
| 0.55 | 0.84 | 0.34 | 0.97 | 0.82 | 0.26 | 0.96 |
| 0.60 | 0.84 | 0.26 | 0.98 | 0.81 | 0.19 | 0.97 |
| 0.65 | 0.83 | 0.19 | 0.99 | 0.81 | 0.14 | 0.98 |
| 0.70 | 0.82 | 0.12 | 1.00 | 0.81 | 0.08 | 0.99 |
| 0.75 | 0.81 | 0.07 | 1.00 | 0.80 | 0.04 | 0.99 |
| 0.80 | 0.80 | 0.02 | 1.00 | 0.80 | 0.01 | 1.00 |
| 0.85 | 0.80 | 0.00 | 1.00 | 0.80 | 0.00 | 1.00 |
| 0.90 | 0.80 | 0.00 | 1.00 | 0.80 | 0.00 | 1.00 |
| 0.95 | 0.80 | 0.00 | 1.00 | 0.80 | 0.00 | 1.00 |
| 1.00 | 0.80 | 0.00 | 1.00 | 1.00 | 0.00 | 1.00 |
